# Biosynthesis approach of zinc oxide nanoparticles for aqueous phosphorous removal: physicochemical properties and antibacterial activities

**DOI:** 10.1186/s13065-023-01012-2

**Published:** 2023-08-16

**Authors:** Mona Khamis, Gamal A. Gouda, Adham M. Nagiub

**Affiliations:** Department of Chemistry, Faculty of Science, Al-Azhar University, Assiut, 71524 Egypt

**Keywords:** Zinc oxide, Nanoparticles, Adsorption, Phosphorus, Antibacterial

## Abstract

In this study, phosphorus (PO_4_^3–^-P) is removed from water samples using zinc oxide nanoparticles (ZnO NPs). These nanoparticles are produced easily, quickly, and sustainably using Onion extracts (*Allium cepa*) at an average crystallite size of 8.13 nm using the Debye–Scherrer equation in the hexagonal wurtzite phase. The characterization and investigation of bio-synthesis ZnO NPs were carried out. With an initial concentration of 250 mg/L of P, the effects of the adsorbent dose, pH, contact time, and temperature were examined. At pH = 3 and T = 300 K, ZnO NPs achieved the optimum sorption capacity of 84 mg/g, which was superior to many other adsorbents. The isothermal study was found to fit the Langmuir model at a monolayer capacity of 89.8 mg/g, and the kinetic study was found to follow the pseudo-second-order model. The adsorption process was verified to be endothermic and spontaneous by thermodynamic characteristics. As a result of their low cost as an adsorbent and their high metal absorption, ZnO NPs were found to be the most promising sorbent in this investigation and have the potential to be used as effective sorbents for the removal of P from aqueous solutions. The antimicrobial activity results showed that ZnO NPs concentration had greater antibacterial activity than conventional Cefotaxime, which was utilized as a positive control in the inhibitory zone. However, no inhibitory zone was visible in the controlled wells that had been supplemented with onion extract and DMSO.

## Introduction

In their natural state, phosphorus is an important nutrient in aquatic habitats (P). It frequently results in eutrophication, a threat to the ecosystem's health, and creates immediate and long-term aesthetic and environmental problems in lakes, reservoirs, bays, coastal areas, and other small bodies of water. This element is often present in small amounts as phosphate ions (PO_4_^3–^). Man-made substances like detergent, fertilizer, pesticides, additives, home, and industrial sewage, and inadequately treated wastewater are to blame for high phosphate levels [1–4]. This excessive rate of (PO_4_^3–^) can damage the natural food chain by promoting the growth of algae through eutrophication and decreasing the amount of dissolved oxygen. Aquatic species may be killed as a result, and the quality of water reservoirs may also suffer [5]. Sewage dumping into waters has grown since 1970. P was thus designated as a significant contaminant due to its large contribution to the eutrophication and diversion of the receiving waters. The EPA recommends that a stream's total P concentration not exceed 0.05 mg/L [6]. Therefore, before waste effluents are released into the environment, it is extremely important to improve phosphate removal [7, 8]. The need to create more effective solutions for the treatment of wastewater that contains PO_4_^3–^ ions arises from the fact that these ions are challenging to remove using existing wastewater treatment methods.

The main phosphate removal methods used today include chemical precipitation using ferric or aluminum salts [9, 10], ion exchange [11], biological removal [12], and adsorption [13, 14] are the principal treatment strategies used to remove phosphate. These technologies, however, typically are unable to cost-effectively and fully comply with the increasingly strict requirements on the release of phosphate. Further, the world's supply of cheap phosphorous, a valuable nonrenewable natural resource, is predicted to run out by 2050 [15]. Investigating workable solutions to capture, recover, and reuse the phosphorus in wastewater is therefore essential. Adsorption appears to be appealing for removing of phosphate due to its ease of use, low cost, ability to regenerate the adsorbents employed for numerous purposes (via desorption), flexibility in design and operation, and cost-effective phosphate recovery [16].

The biological synthesis of NPs can be carried out using a vast array of resources such as plants and plant products, algae, fungi, yeast, bacteria, and viruses. Nanoparticles produced by plants are more stable and the rate of synthesis is faster than in the case of microorganisms. Furthermore, plants are eco-friendly [17], sustainable [18], free of chemical contamination, less expensive [19], and can be used for mass production [20]. Furthermore presence of various compounds, such as proteins, alkaloids, flavonoids, reducing sugars, polyphenols, etc., in the biomaterials act as reducing and capping agents for the synthesis of NPs from its metal salt precursors [21]. Phytochemical profile of the onion extract is mainly derived from the carbohydrate, lipid, protein, and polyphenols moieties, also quercetin and quercetin 4'-O-β-glucopyranoside were involved in the formation of metal oxide nanoparticles [22].

**Fig. 1:**
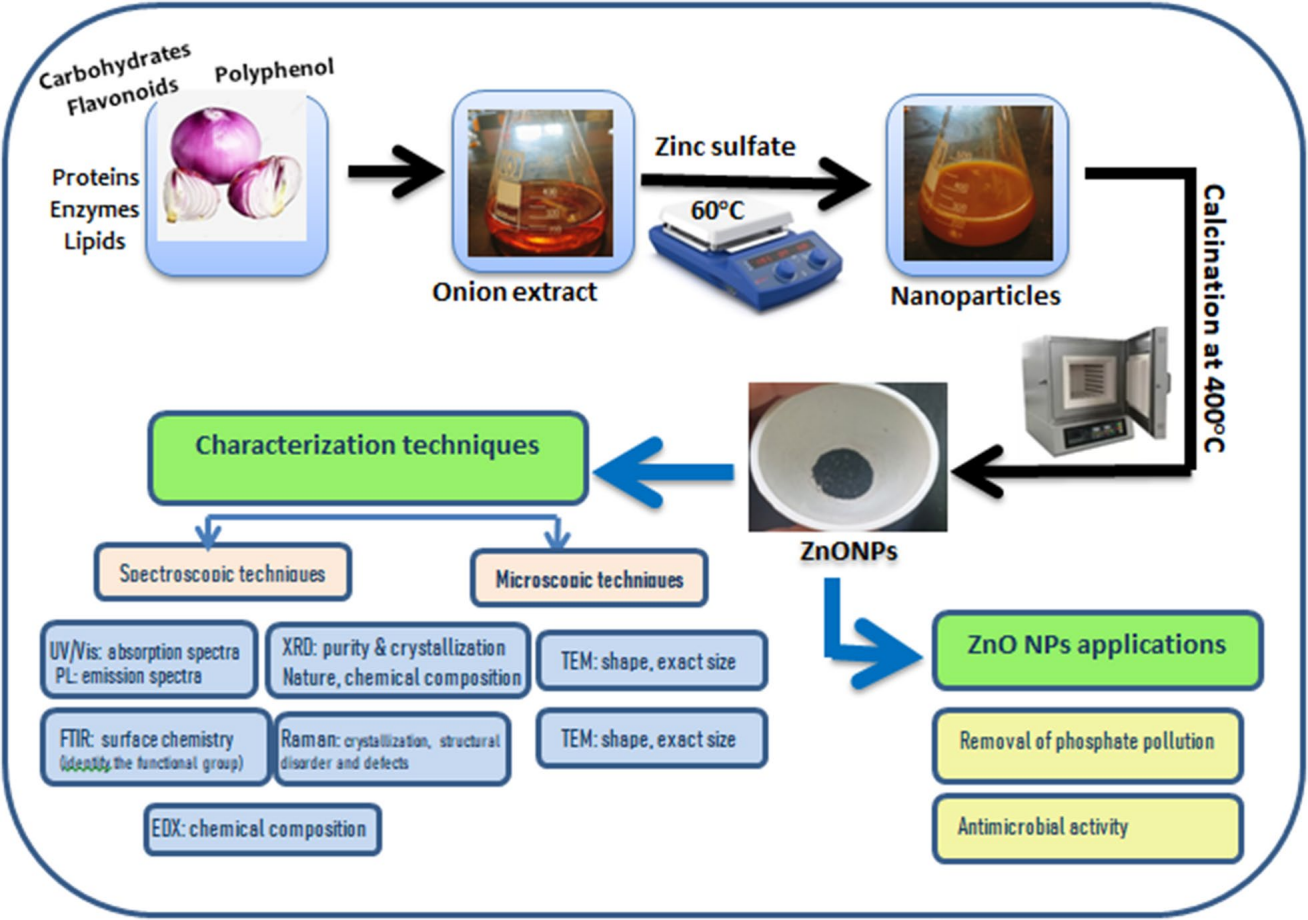
Schematic representation of biosynthesized ZnO NPs, characterization techniques, and applications

Here, a green method for producing ZnO nanoparticles using onion extract has been established. Then, physicochemical characterization was carried out using various techniques to assist the nanoscale biosynthesis of ZnO NPs. It investigated how well ZnO NPs achieved unprecedented removal of phosphorus from wastewater through adsorption, and evaluate of reusing and regeneration efficiency of prepared nanomaterials. Furthermore, the antibacterial properties of ZnO NPs were investigated against Gram-positive and Gram-negative bacteria. A schematic representation of green synthesis of ZnO NPs and the goal of the study (Fig. 1).

## Experimental work

### Materials and reagents

Except as noted in work, all the chemicals and materials used in this work were obtained in their original states. Sigma-Aldrich provided zinc sulfate heptahydrate (ZnSO_4_.7H_2_O) with a purity of 98% and potassium dihydrogen phosphate (KH_2_PO_4_) with a purity of 99%. By mixing 4.0 g of sodium hydroxide (NaOH) extra pure (LOBA CHEMIE) with 600 mL of deionized water, 1 Liter was created. A 37% quantity of hydrochloric acid (HCl) was purchased from Merck. Cefotaxime [C_16_H_17_N_5_O_7_S_2_], 455.46 g/mol, serving as an antimicrobial standard, was supplied by EVA Pharma, Egypt.

### Green synthesis of ZnO NPs

A small amount of the red onion plant about 100 g was thoroughly cleaned with distilled water before being shade-dried at room temperature and steeped in 50% aqueous ethanol (50:50 v/v) in a glass tank. After 60 h, the solid material is eliminated by being filtered twice with Whatman filters to eliminate any remaining solids. Heat 100 mL of onion extract using a hotplate magnetic stirrer to 60 °C before adding 60 mM of aqueous zinc sulfate dropwise. Magnetic stirring was used to thoroughly combine the solution. Immediately after addition, a brown precipitate was seen. The pH was then adjusted to 10.5 by adding a few drops of 25% ammonia solution and keeping the temperature at 60 °C while constantly stirring. The precipitate is clearly distributed throughout the solution, allowed to react for one hour and stand for 24 h. This precipitate was periodically rinsed with distilled water, centrifuged at 5000 rpm for 20 min to remove the ethanol, and then dried for 6 h at 60 °C in the oven. The dry precipitate was annealed for two hours at varied temperatures between 200 and 400 °C in an open furnace. The best process for producing extremely crystalline ZnO nanoparticles was annealing at 400 °C.

### Instrumentation

The production of ZnO NPs was measured using a digital balance model 220 (Denver Instrument Co., USA), a heater magnetic stirrer 1L (0–1600 RPM), and an electric muffle furnace (ST-1200 °C-666, Germany). Using an X-ray diffractometer (PW 1710 from Philips), electron microscopy (QUANTAFEG 250 with an EDX attachment), and transmission electron microscopy (TEM28 JEOL JEM-100C XII), the size of the ZnO NPs was determined. Using a PerkinElmer (Lambda 750 UV/Vis/NIR) Spectrophotometer, the samples' UV–Vis absorption spectra measurements were taken in the wavelength range of 200–800 nm. Using a Thermo Fisher Scientific device, the FT-IR spectra were captured in the wavenumber range 4000–400 cm^**–**1^ (Nicolet iS10 FT-IR Spectrometer). On the Hitachi, photoluminescence measurements (PL) were carried out (F-7100 Fluorescence Spectrophotometer). Using a micro-Raman Horiba Jobin Yvon LabRam spectrometer, Raman analysis was carried out (HR800).

### Adsorption studies

KH_2_PO_4_ was dissolved in distilled water to produce a solution containing P (1000 mg/L). Dilution was used to create the working concentrations that were required. The pH of the solution was then adjusted using 0.1 M NaOH and HCl solution to the required value. For the batch sorption studies, the pH of the solution was increased from 3 to 9, and the contact time and temperature were set at 0–1440 min and 20–40 °C, respectively. The studies were conducted using a solution with an initial P ions concentration of 5–400 mg/L and a nanoparticle dose of 0.015 − 0.045 g/L. ZnO NPs were used in the adsorption studies in 100 mL stoppered conical flasks with 20 mL of P ions solutions. The pH was then corrected, and the flasks were then put into a shaker at a 120 rpm speed. The following filtration to remove the adsorbent, the concentration of the remaining P ions was determined spectrophotometrically at 400 nm using the vanado molybdous phosphoric acid technique.

#### *Point of zero charge (pH*_*pzc*_*) and effect of initial pH adsorption*

The pH drift method was used to determine the pH of the point of zero charges (pH_pzc_), or the pH below which a nanoparticle's entire surface is positively charged. To do this, 20 mL of 0.1 mol/L NaCl solutions were created with initial pH ranges of 2–10, 0.1 N HCl, or 0.1 N NaOH, and 0.025 g of ZnO NPs. The samples were then agitated for 24 h, and the pH stabilized for 48 h at room temperature. The mixture's final pH was measured, and the final pH (Y-axis) was plotted against the initial pH to demonstrate their differences (X-axis). This curve's intersection with the initial pH = final pH line is known as pH_zpc_ [23]. The studies were conducted in the pH range of 3.0–9.0 while maintaining the following constants: P ions concentration of 250 mg/L, adsorbent dose of 0.04 g, contact period of 24 h, and temperature of 27 °C. The pH of the solution was adjusted to the required level using 0.1 M HNO_3_ or 0.1 M NaOH.

#### Effect of adsorbent dosage

By varying the amounts of the adsorbents in the test solution from 0.015 to 0.045 g/L while maintaining the initial P ions concentration at 250 mg/L, the temperature at 27 °C, pH 3.0, and equilibrium time of 24 h, the adsorption of P ions onto ZnO NPs was examined.

#### Isothermal study and effect phosphorous concentration

Aqueous solutions of P ions in the concentration range of 50–400 mg/L were generated to thoroughly grasp the nature of the interaction and to characterize how the adsorbate interacts with adsorbents. Langmuir, Freundlich, Temkin, and Dubinin-Radushkevich (D-R) isothermal models were used to assess the equilibrium data for the adsorption of P ions onto produced ZnO NPs [24], as the following equations:

1 $${\text{P}}\,{\text{adsorbed}}\,{\text{amount}}\,q_{e } \, = \,\left( {C_{0} \, - \,C_{e} } \right)\,V\,/\,m$$

2 $${\text{P}}\,{\text{adsorption}}\,\left( \% \right)\, = \,\left( {C_{0} \, - \,C_{e} } \right)\,/\,C_{0 } \, \times \,100$$

where V is the volume of the P (mL), q_e_ is the amount of P that has been adsorbed (in mg/g), C_0_ is the starting concentration of P (in mg/L), C_e_ is the equilibrium concentration of P (in mg/L), and m is the mass of ZnO NPs (mg).

3 $$C_{e} /q_{e } \, = \,\left( {1/q_{L} K_{L} } \right)\, + \,\left( {1/q_{L} } \right)C_{e}$$

4 $$R_{L} \, = \,1/\left( {1\, + \,K_{L} C_{max} } \right)$$

where q_L_ is the monolayer adsorption capacity of ZnO NPs (mg/g), K_L_ is Langmuir energy of adsorption constant (L/mg), R_L_ is the sensitive equilibrium parameter and C_max_ is the highest initial P concentration in the solution (mg/L).

5 $$log\,q_{e} \, = \,log\,K_{F} \, + \,\left( {1/n} \right)\,log\,C_{e}$$

K_F_ is the Freundlich adsorption capacity of ZnO NPs (mg/g), and n is the Freundlich constant, indicating the adsorption intensity.

6 $$q_{e } \, = \,B_{T} ln\,A_{T} \, + \,B_{T} ln\,C_{e}$$

7 $$b_{T} \, = \,RT/B_{T}$$

where A_T_ is the binding constant (L/mg), which was related to the maximum binding energy. The B_T_ is the Temkin adsorption constant (KJ/mol) related to the sorption heat, R is the gas constant (8.314 J/mol K), T is the absolute temperature at 298 K and b_T_ is the adsorption process constant.

8 $$ln\,q_{e } \, = \,ln\,q_{m} \, - \,\beta \varepsilon^{2}$$

9 $$\varepsilon \, = \,RT\,\left( {1\, + \,1/C_{e} } \right)$$

10 $$E_{D} \, = \,\left( { - \,2\,\beta } \right)^{{{-}1/2}}$$

where q_m_ is the D-R adsorption capacity of ZnO NPs (mg/g), β is the coefficient related to the mean free energy, ε is Polanyi potential and E_D_ is adsorption energy per molecule of the P adsorbate when it is transferred to the surface of the solid ZnO NPs from infinity in the solution (kJ/mol).

#### Kinetic and thermodynamic studies

By adding 0.04 g of the nano sorbent to a 100 mL conical flask containing 20 mL of the phosphorous solution (250 mg/L) at a constant temperature of 300 K, P is removed by ZnO NPs as a function of contact time. For various contact times, the P ion residual concentration was calculated (from 0 min to 24 h). The following Eqs. (11–14) were used to assess the adsorption data toward phosphorous following the kinetic models:

Pseudo-first-order model [25]:

11 $$log\,\left( {q_{e } \,{-}\,q_{t} } \right)\, = \,log\,q_{e} \,{-}\,\left( {K_{1} /2.303} \right)\,t$$

Pseudo-second-order model [26]:

12 $$\left( {t\,/\,q_{t} } \right)\, = \,1\,/\,\left( {K_{2} q_{e}^{2} } \right)\, + \,\left( {1\,/\,q_{e} } \right)\,t$$

where q_t_ represents the amount of P contaminant adsorbed by ZnO NPs (mg/g) at a predetermined time interval t, K_1,_ and K_2_ are rated constant of pseudo-first-order and pseudo-second-order adsorption process respectively, (min^–1^).

Elovich model [27]:

13 $$q_{t} \, = \,(1\,/\,\beta )\,(ln\alpha \beta )\, + \,(1\,/\beta )\,ln\,t$$

where β is the constant related to surface coverage and the activation energy for chemisorption (g/mg), and α is the initial sorption rate constant (mg/g min).

Weber’s and Moris’s intraparticle diffusion model [28]:

14 $$q_{t} \, = \,C\, + \,K_{int} \,\left( t \right)^{1/2}$$

where K_int_ is the intraparticle rate constant (mg/g min^1/2^), and C is the value that gives information about the boundary thickness.

Equations 15–17 can be used to determine the parameters Gibbs free energy change (ΔG) (kJ/mol), enthalpy change (ΔH) (kJ/mol), and entropy change (ΔS) (kJ/mol K) to assess the thermodynamic behavior of phosphorus adsorption on ZnO NPs [29]:

15 $$\Delta G\, = \, - \,RT\,ln\,K_{c}$$

16 $$ln\,K_{c} \, = \, - \,\Delta G\,/\,RT\, = \, - \,\left( {\Delta H\,/\,RT} \right)\, + \,\left( {\Delta S\,/\,R} \right)$$

17 $$K_{c} \, = \,C_{ads} \,/\,C_{e}$$

∆G determines the free energy change (J/mol), R is the gas constant (8.314 J/mol K), T is the absolute temperature (K), and K_c_ is the thermodynamic equilibrium constant, ∆H determines the enthalpy change (J/mol), ∆S represents the entropy change (J/mol K) and C_ads_ acts the concentration (mg/L) of the adsorbed P.

### Recovery of phosphorous and reuse adsorbent experiments

The dilute sodium hydroxide is used to elute the nano-adsorbents loaded with phosphorus ions, and the pH is subsequently adjusted with deionized water washings. The regeneration experiment was then carried out for four cycles, dried at 70 °C to constant weight, and re-loaded with P ions to examine the lifespan of the nano-adsorbents and removal percentage. After filtering the suspension from the adsorption test, the evaluated phosphorus desorption from the sorbent was collected in a 250 mL Erlenmeyer flask. 100 mL of a phosphate-free solution was added to each flask. After that, the flask was shaken for 24 h while the pH was maintained within a wide range. In a manner comparable to previously reported, the suspension solutions underwent filtering and phosphorus desorbed analysis. The amount of phosphate in the solution following the desorption experiment was used to calculate the amount of desorbed phosphorus.

### Antimicrobial activity

In this study, we use the good diffusion method to examine the antibacterial properties of green-produced ZnO NPs against different microorganisms. *Salmonella typhimurium* ATCC 14028 and *Escherichia coli* ATCC 25922 were the bacteria employed for the antibacterial activity, whereas *Enterococcus faecalis* ATCC 29212 and *Staphylococcus aureus* ATCC 25923 were the Gram-positive bacteria. The Central lab provided bacterial strains. Egypt's Water Resources and Irrigation Ministry is in charge of the New Valley governorate.

Cefotaxime, a common antibacterial drug, was utilized as a positive control, while DMSO was used as the solvent. 50, 100, 150, and 200 μg/mL of ZnO NPs were produced at four different concentrations. Gram-positive and Gram-negative bacteria were treated with four different concentrations in DMSO in a 50 μL. The tests were then conducted using Cefotaxime 150 μg/mL as an antimicrobial standard (positive control), with an *Onion* extract in an aqueous liquid and DMSO as the negative control. The experiment was run in triplicates, and each plate's zone of inhibition was quantified. Before the experiment, pure cultures were sub-cultured on nutrient broth, incubated at 37 °C for 24 h, and then diluted into the same broth to a concentration of about 10^–6^ cfu/mL. Poured nutrient agar over each culture diluted to 1 mL and transferred to the plate. Sterilized pipette tips were then used to create the wells. Transferred from (50, 100, 150, and 200 μg/mL) of ZnO NPs, a 50 μL was put into wells. The plates were then allowed to stand for diffusion before incubating for 24 h at 37 °C.

## Results and discussion

### XRD analysis

**Fig. 2:**
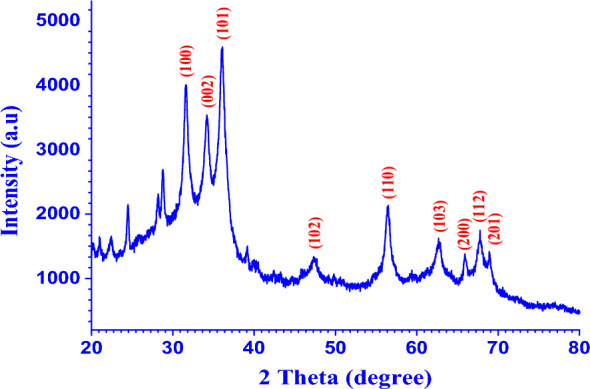
XRD of biofabricated ZnO NPs produced by onion extract

The XRD pattern of ZnO NPs generated using the biosynthetic process is shown in Fig. 2. Diffraction peaks were observed at 31.61°, 34.19°, 36.11°, 47.32°, 56.49°, 62.67°, 67.77° 65.85°, and 68.9° corresponding to lattice planes (100), (002), (101), (102), (110), (103), (200), (112), and (201) respectively. With JCPDS card No. 01–079-0208, the XRD patterns of ZnO NPs are indexed, indicating the typical hexagonal wurtzite phase [30]. The fact that reflection (101) had the highest intensity of all the patterns revealed that most of the particles were pointed in that direction. The large and prominent peaks in Fig. 2 demonstrate strain in the particles and the production of smaller zinc oxide nanoparticles [31]. The onion species, which is capping and stabilizing the nanoparticles, is responsible for the other diffraction peaks, seen at 24.48, 28.76, and 40.5 degrees.

The Debye–Scherrer method was also used to estimate the average particle size of ZnO NPs (Eq. 18). The average particle size is determined to be 8.13 nm, which is compatible with the findings of SEM and TEM [32, 33]. The structural and microstructural parameters were estimated, including lattice parameters, lattice spacing, respective reflections, and particle sizes [34]. Equations 18–22 were used to get the unit cell volume for examples and the lattice parameters [34]:

18 $$D\, = \,0.9\lambda \,/\,\beta cos\left( \theta \right)$$

D determines the average crystallite size, K is constant (0.94), λ is the wavelength (Cu-K_α_ = 1.54171 Å), Ɵ is the Bragg angle, and β: The full width at half maxima (FWHM) of a diffracted peak.

19 $${\varvec{a}}\, = \,\frac{{\varvec{\lambda}}}{{\sqrt 3 \user2{ }\sin {\varvec{\theta}}_{{\left( {100} \right)}} }}$$

20 $${\varvec{c}}\, = \,\frac{{\varvec{\lambda}}}{{\user2{sin\theta }_{{\left( {002} \right)}} }}$$

21 $${\varvec{v}}\, = \,\frac{\sqrt 3 }{2}\,{\varvec{a}}^{2} {\varvec{c}}$$

22 $${\varvec{D}}^{{\varvec{x}}} \, = \,\frac{{16{\varvec{M}}}}{{{\varvec{Na}}^{2} }}$$

where c/a is valuing confirmed that the prepared NPs were crystalline, v is unit cell volume (Å)^3^, D^x^ is X-ray density, M is molecular mass, and N is Avogadro’s number (6.0223 × 10^23^ particles mol^–1^).

The calculated values of a and c, shown in Table 1, accord well with the reported values (a = 3.246, c = 5.219, JCPDS card number 0208–079-01).

**Table 1:** Comparison between XRD results of biosynthesized ZnO NPs and standard ZnO powder

ZnO NPs	a (Å)	c (Å)	v (Å^3^)	c/a	D^x^ × 10^4^ (kg m^−3^)
Standard	3.264	5.219	48.17	1.598	6.213
Sample	3.2521	5.2221	47.83	1.604	6.214

### FT-IR analysis

**Fig. 3:**
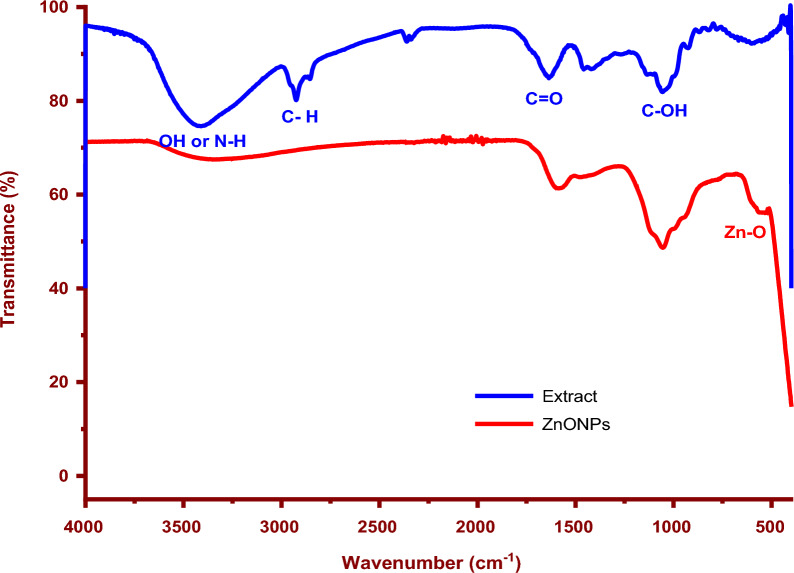
FT-IR spectra of biofabricated ZnO NPs and onion extract

The FT-IR spectra of onion extract and biosynthesized ZnO NPs are shown in Fig. 3. The bands at 3407, 2927, 1636, 1450, and 1055 cm^−1^ show the onion extract's complex phytochemical composition, which is predominantly produced by the carbohydrate, protein, lipid, and polyphenol [35]. The peak at 3407 cm^−1^ is due to the alcohol and phenolic groups being stretched O − H, proteins being stretched N − H, and water being adsorbed [36, 37]. The peak at 2927 cm^−1^ is attributed to asymmetric stretching of C − H. Carbonyl vibrations in the onion extract may have contributed to the creation of ZnO NPs and are responsible for the characteristic peak at 1636 cm^−1^ [4, 38, 39].

For instance, the bands at 1585 cm^−1^ belonging to the C = O group changed to a lower frequency area, while the broad peak at 3319 cm^−1^ related to O–H or N–H had previously been detected in plant extract grew broad [40]. The bands at 1055 cm^−1^ were associated with the carboxylic acid functional groups and the C–O stretching of ester. The similarity of the ZnO NPs and onion extract spectra indicates the presence of bioactive organic species as reducing agents, which also shows some shifts. The majority of the signals for various functional groups, as shown in Fig. 3, confirm that these reducing groups are also responsible for forming and stabilizing preparation nanoparticles [3, 41]. The generation of pure ZnO NPs was confirmed by the new sharp and weak peak at 536 cm^−1^, which follows ZnO stretching vibration [42, 43].

### Optical characteristics

**Fig. 4:**
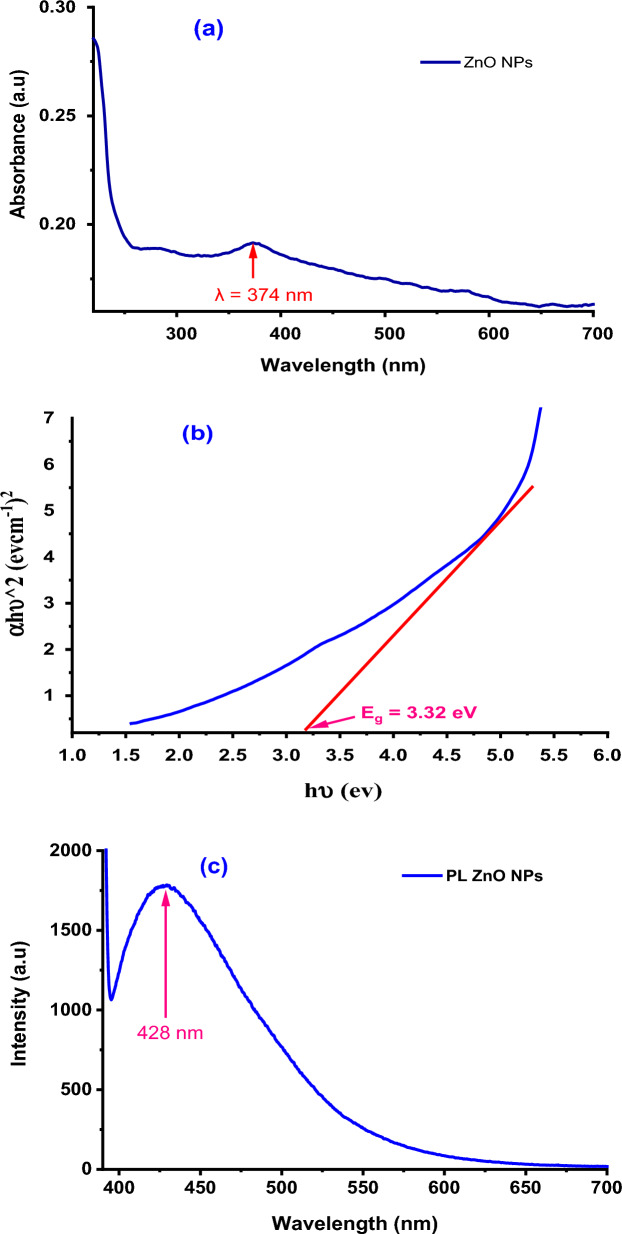
UV–vis absorption (**a**), plots of (αhѵ)^2^ as a function of photon energy hʋ for a direct bandgap of ZnO NPs (**b**) and PL spectra of ZnO NPs showed the emission peaks at 428 nm (**c**)

Figure 4a displays the ZnO NPs distributed in water's UV–Vis absorbance. The distinctive peak for hexagonal wurtzite ZnO is at 374 nm, and this is quite similar to how ZnO NPs are made by Parthenium leaf extract [44]. Because shallow levels have developed, the absorption peak has a red shift of roughly 9 nm relative to that of bulk ZnO (365 nm) [45, 46]. Additionally, no additional peaks in the spectrum were seen outside the typical peak, proving the great purity of ZnO NPs made from onion extract. The electronic band gap of semiconductors can be calculated using Eq. 23 of the Tauc relationship [44].

23 $$\left( {\user2{\alpha h\nu }} \right)^{{\varvec{n}}} \, = \,{\varvec{A}}\left( {\user2{h\nu } - {\varvec{E}}_{{\varvec{g}}} } \right)$$

where α is the absorption coefficient (α = 2.303 A/t; A is the absorbance and t is the cuvette thickness), h is the Planck’s constant, ν is the photon frequency, n value is 2 for direct band gap semiconductor and E_g_ is the optical band gap.

By plotting (αhѵ)^2^ vs the photon energy hѵ using the information from the absorption spectra, as shown in Fig. 4b, it is possible to determine the optical energy gap, or E_g_, of the ZnO NPs. It demonstrates that in a specific area, the resultant plotting provides tangent to the linear section of the curves. ZnO NPs produced through biosynthesis have an estimated band gap of 3.32 eV [47, 48]. Figure 4c displays the results of the photoluminescence spectrum (PL) analysis to look into the crystal flaws. The sample found in the blue-green zone, clearly showed the deep-level emissions. The presence of inherent crystal flaws in the ZnO NPs was blamed for the blue-green emission bands seen at 428 nm (excitonic transitions) [34].

### Raman analysis

**Fig. 5:**
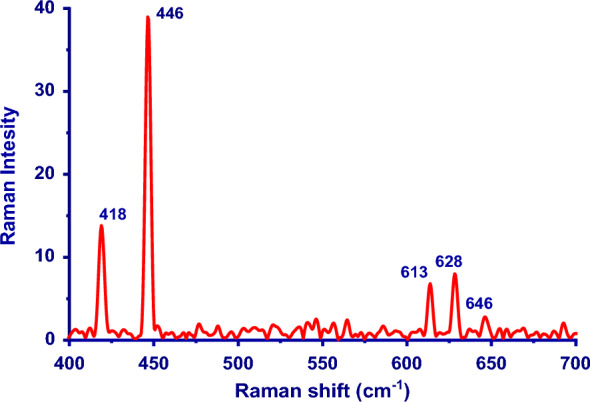
Raman spectrum of biosynthesis ZnO NPs

ZnO nanoparticles having a wurtzite structure that is hexagonal fit into the P63mc space group [49]. Only the optical phonons near the Brillouin zone's point Γ, participate in first-order Raman scattering for the ideal ZnO crystal. Equation 24 lists the optical modes that should be present in a wurtzite ZnO based on the group theory.

24 $${\varvec{\varGamma}}_{{{\varvec{opt}}}} \, = \,{\varvec{A}}_{1} \, + \,2{\varvec{B}}_{2} \, + \,{\varvec{E}}_{1} \, + \,2{\varvec{E}}_{2} \user2{ }$$

where, both A_1_ and E_1_ modes are polar branches, split into a transverse optical mode (TO), and a longitudinal optical mode (LO). E_2_ mode consists of low E_2_^low^ and high E_2_^high^ frequency phonons modes. E_2_^low^ is associated with the vibration of the oxygen atom and E_2_^high^ is related to heavy Zn sublattices [50]. The first-order Raman-active modes are A_1_, E_1_, and E_2_. Additionally, the B_1_ modes, also known as silent modes are typically inactive in Raman spectra. Figure 5 shows the Raman spectra for the ZnO NPs, which had typical peaks in the 400–700 cm^−1^ range. One of the distinctive modes of the hexagonal wurtzite phase of zinc oxide, the high-frequency branch of the nonpolar optical phonon E_2_^high^ of zinc oxide matched to the high-intensity peak seen at 446 cm^−1^. The high-intensity peak's correlation with the E_2_^high^ optical mode also emphasized the remarkable structural integrity and optical properties of the greenly synthesized ZnO NPs made from onion extract [51]. The additional peaks at 613 and 628 cm^−1^ do not match up with ZnO normal modes. These peaks represent other vibrational modes linked to faults [52].

### Morphology analysis (SEM, TEM, and EDX analyses)

**Fig. 6:**
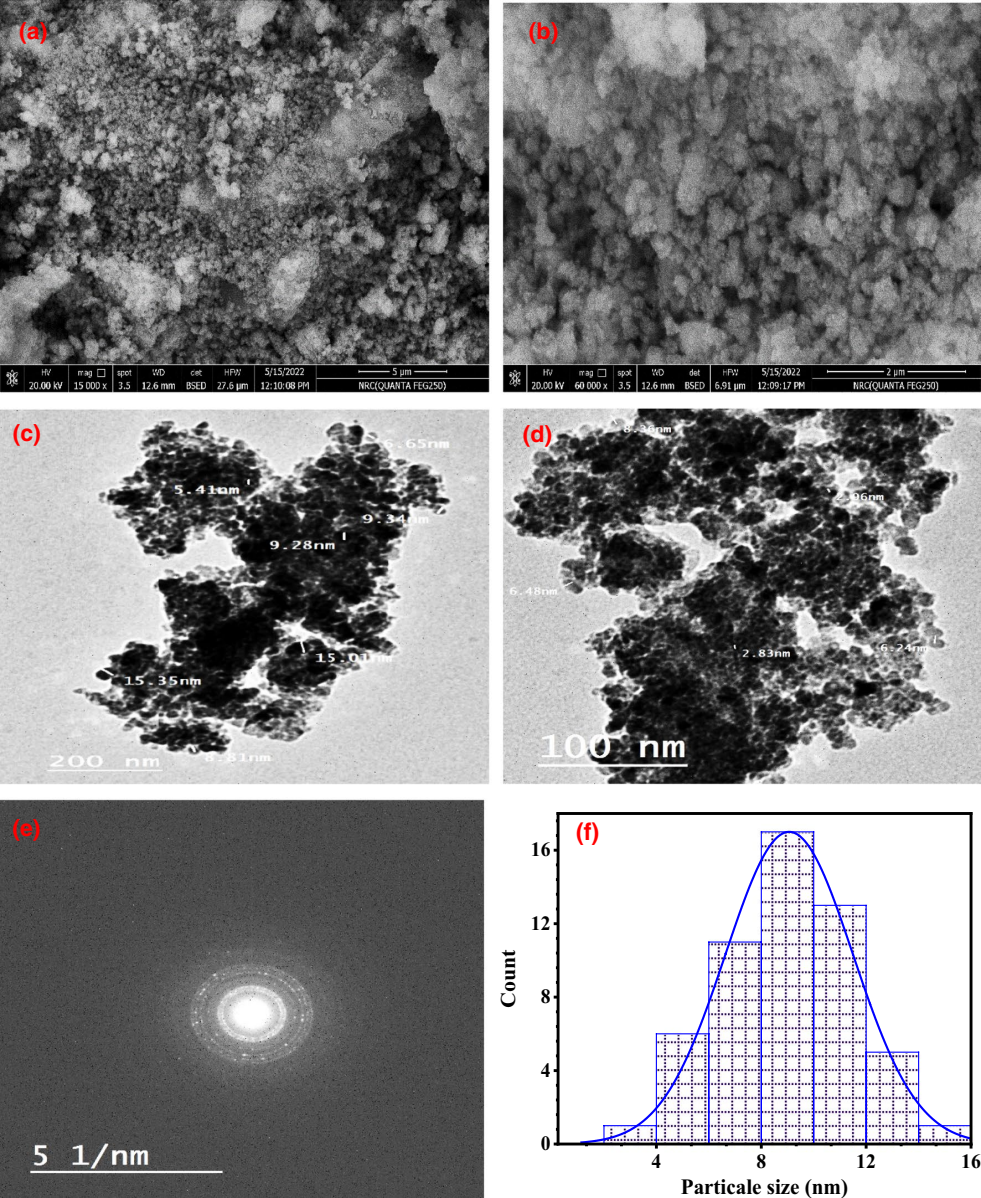
Characterization of the biosynthesized ZnO-NPs: **a**, **b** represents the SEM image, **c**, **d** denotes the TEM image, **e** SAED pattern and **f** particle size distribution

The exterior morphology of ZnO NPs is depicted in Fig. 6a and b as having a spherical form and high aggregation. The particles were found to be aggregated, and this kind of accumulation resulted from the produced nanoparticles' high surface energy [53]. The biomimetically synthesized ZnO NPs were produced from onion extract, and SEM pictures showed that they are smaller than previously described (Table 2). The spherical ZnO NPs with a typical size of 2.83 to 15.35 nm are shown by the TEM investigation of the bio-synthesized NPs in Fig. 6c and d. Figure 6e shows that the biosynthesized ZnO-NPs were polycrystalline in nature. The smallest particles in the sample ranged 1 to 16 nm, with an average diameter of 8.89 nm, as can be seen from particle size distributions Fig. 6f. The observed size is in good agreement with the XRD result. When the pH is raised throughout the synthesis process, thin, spherical nanoparticles become aggregates of tiny clusters, as shown in TEM pictures (until pH 10) [54, 55].

**Table 2:** Comparison between the particles size of ZnO NPs prepared from onion extract and some other plants

No	Plant source	Part extracted	Resource of ZnO	Size	References
1	*C. sinensis*	Peel	Zinc nitrate	22.6 nm	[57]
2	*Moringa oleifera*	Leaves/seeds	Zinc acetate dihydrate	10.8–13.2 nm	[58]
3	*Coriandrum sativum*	Leaves	Zinc acetate dihydrate	9–18 nm	[59]
4	*Ferulago angulata*	–	Zinc acetate dihydrate	32–36 nm	[60]
5	*Coriandrum sativum*	Leaves	Zinc acetate	24 nm	[61]
6	*Cyanometra ramiflora*	Leaves	Zinc acetate	13 nm	[41]
7	*Oak*	Fruit hull	Zinc acetate dihydrate	34 nm	[62]
8	*Garcinia mangostana*	Fruit	Zinc nitrate hexahydrate	21 nm	[63]
9	*Mussaenda frondosa*	Leaves/stem	Zinc acetate dihydrate	5–20 nm	[64]
10	*Vitex trifolia L*	Leaves	Zinc nitrate hexahydrate	28 nm	[65]
11	*Sambucus ebulus*	Leaves	Zinc acetate dihydrate	17 nm	[66]
12	*Tabernaemontana divaricata*	Green Leaves	Zinc nitrate	20–50 nm	[67]
13	*Dolichos lablab L*	Leaves	Zinc acetate dihydrate	_29 nm_	[68]
14	*Carica papaya*	Latex	Zinc nitrate	11–26 nm	[69]
15	*Betel*	Leaves	Zinc acetate	50 nm	[70]
16	*Azadirachta indica*	Leaves	Zinc nitrate	9–38	[70]
17	*Cannabis sativa*	Leaves	Zinc acetate	38 nm	[71]
18	*Sea buckthorn*	Fruit	Zinc nitrate hexahydrate	_17.15 nm_	[72]
19	*Chlorella*	–	Zinc nitrate	20 ± 2.2 nm	[73]
20	Onion extract	Fruit	Zinc sulfate	2.06–15.3 nm	This study

**Fig. 7:**
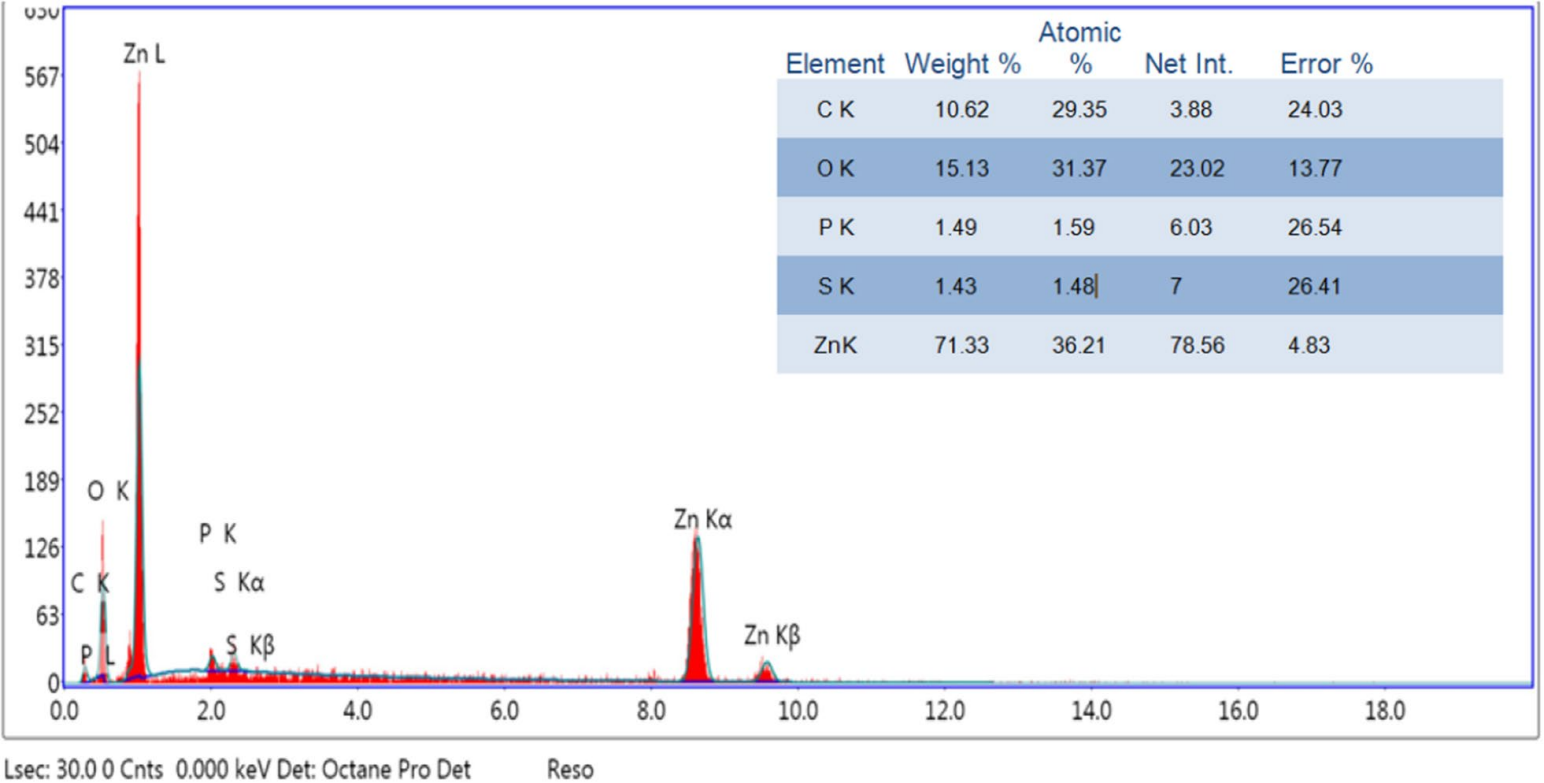
Analysis of the chemical composition of bio-synthesized ZnO NPs

To determine the elemental composition of the produced ZnO NPs, an energy-dispersive X-ray diffractive (EDX) analysis was conducted. The sample made using the above technique has only pure ZnO phases, according to SEM–EDS examination of the ZnO NPs. As shown in Fig. 7, the EDX validates the presence of zinc and oxygen signals in ZnO NPs. This analysis revealed the distinct peaks for both Zn and O. Additionally, measurement of the components revealed that the sample included 71.33% Zn and 15.13% O by weight, demonstrating that the created ZnO NPs are in their purest form [43]. While the other minor constituents present in the ZnO NPs were due to the componants of the onion plant extract [56].

### Phosphorus wastewater remediation via ZnO NPs (batch adsorption examination)

#### Zero point charge and effect of initial pH adsorption

**Fig. 8:**
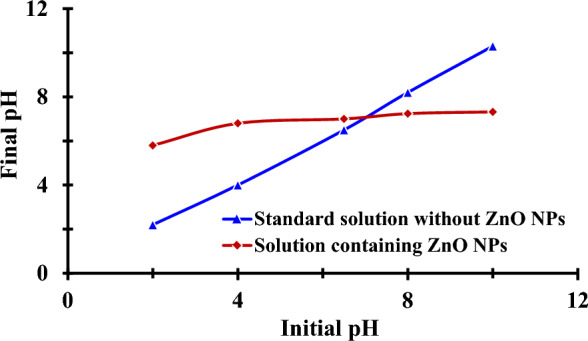
Zero-point charge (pH_zpc_) of the biofabricated ZnO NPs

Figure 8 depicts the curve for calculating of the pH_zpc_ of ZnO NPs. This diagram makes it clear that the pH range between 4 and 5 goes from acidic to basic, whereas the pH range between 6 and 10 goes from more basic to less basic. The pH_zpc_ of the nanoparticles is 7 because the pH curve of the nanoparticles crosses the straight line at this point.

The following reactions demonstrate how the phosphoric acid could be dissociated into several ionic species such as H_2_PO_4_^−^, HPO_4_^2−^, and PO_4_^3−^ depending on the pH of the solution (Eq. 25):

25 $$H_{3} PO_{4} \,\overset {pK_{1} } \longleftrightarrow \,H_{2} PO_{4}^{ - } \, + \,H^{ + } \,\overset {pK_{2} } \longleftrightarrow \,HPO_{4}^{2 - } \, + \,2H^{ + } \,\overset {pK_{3} } \longleftrightarrow \,PO_{4}^{3 - } \, + \,3H^{ + }$$

where pK_1_ = 2.15, pK_2_ = 7.20 and pK_3_ = 12.33, respectively [74].

**Fig. 9:**
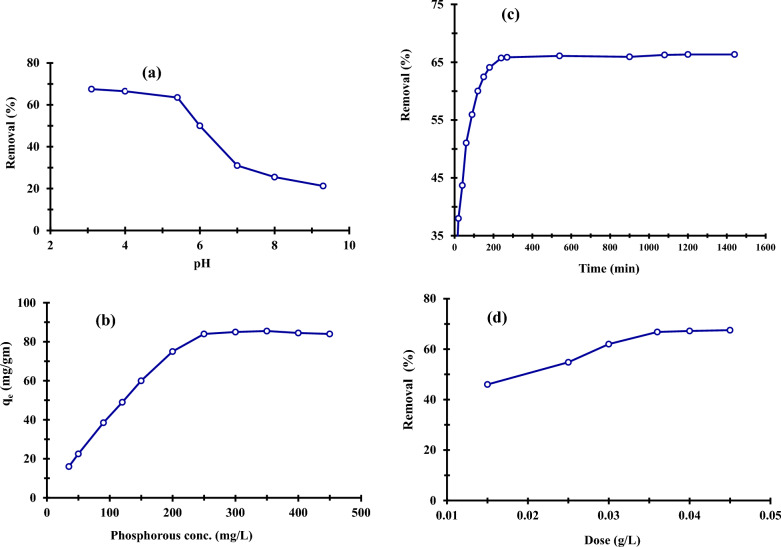
Effect of pH (**a**), the effect of initial phosphorous concentration (**b**), contact time (**c**), and the effect of the dosage of ZnO NPs on the adsorption of P ions

The pH of the solution, which affects the adsorption behavior of phosphorous and is connected to the effect of the adsorbent's pH point of zero charges, therefore determines the protonation/dissociation state of the phosphate hydroxyl groups [75]. The pH rose due to the surface hydroxyls being deprotonated, the surface charge switching from positive to negative, and the quick decrease in the electrostatic contact between P ions and the sorbent surface. Figure 9a illustrates phosphorus removal rate from aqueous solutions onto ZnO NPs based on pH variations (3.0–9.0 pH) at the beginning phosphorus concentration of 250 mg/L, the absorbent dose of 0.04 g/L, contact time of 24 h, and temperature of 27 °C. The pH of the solution, which influences the adsorbent's surface charge, degree of ionization, and speciation, was found to be a significant determinant of the removal of P ions from water by producing adsorbent ZnO NPs [76]. It was shown that when the pH of the solution rose, the NP's adsorption ability decreased, reaching its maximum at low pH levels. At pH 3.1 and 67.5%, the most excellent clearance rate for ZnO NPs was noted. ZnO NPs exhibited a maximum sorption rate of 84 (mg/g). All other adsorption trials in this work used pH = 3.1 because that was the pH at which adsorption occurred at its highest rate. The increased phosphorus removal rate at lower pH levels may be caused by the development of more positive active sites on the nano adsorbents surface, which aids the sorption for generating an electrostatic connection between P ions and the sorbent surface [77]. The electrostatic force and the interplay of ion exchange may explain the removal of P ions from water [78].

#### Effect of initial phosphorous concentration

Figure 9b shows the effect of phosphorus content on the produced adsorbent's removal capability at pH 3.0, 40 mg of adsorbent dose, 27 °C, and 24 h. The adsorption capabilities nearly remained constant after exceeding 250 mg/L. This may have been caused by insufficient active sites that might hold more P atoms. ZnO NPs have an 84 mg/g maximal adsorption capacity for P ions.

#### Effect of contact time

Adsorption studies were conducted with an initial phosphorus content of 250 mg/L, an adsorbent dosage of 40 mg/L, a solution pH of 3.0, and room temperature during a range of contact periods from 0 to 1440 min (27 °C). Results from Fig. 9c showed that as contact time was prolonged from 10 to 240 min, phosphorous adsorption capacity increased quickly, rising from 39.5 mg/g to 84 mg/g (equivalent to a phosphorous removal rate rise from 31.6 to 66.4%). The adsorption process in this experimental setup slowed after 240 min and steadied at 270 min. The adsorption capacity and phosphorus removal efficiency peaked at 240 min of contact time, reaching maximum values of 84 mg/g and 66.4%, respectively. The ZnO NPs surface initially had many new binding sites available, which caused a rapid increase in adsorption. Over time, the adsorption process became slower and more stable due to the saturation of active adsorption sites on the ZnO NPs surface [79].

#### Effect of dosage

Different adsorbent doses (0.015 − 0.045 g/L) were employed to examine the impact of ZnO NP dosage on the adsorption of P ions. As can be shown, while utilizing a nano-adsorbent dosage of 0.04 g with 250 mg/L P solution, the maximum adsorption of P ions was 67.5%. According to Fig. 9d, there is an early increase in the P ions removal capacity with the mass ratio of solid/liquid, followed by a prolonged increase at the quantity of 0.04 g. This may be explained by the fact that when the nano-adsorbent dose rose, the effective adsorption active sites on the nanomaterial surface rapidly increased, causing a rapid uptake of P ions from the sorption solution. The absorption efficiency of P ions will nonetheless achieve equilibrium as the nanomaterial dose keeps rising. You could think of it as a saturation point. Any increase in the solid dose after this saturation point just causes the adsorbed layer at the adsorbent surface to become thicker [80–83].

#### Kinetic study

**Fig. 10:**
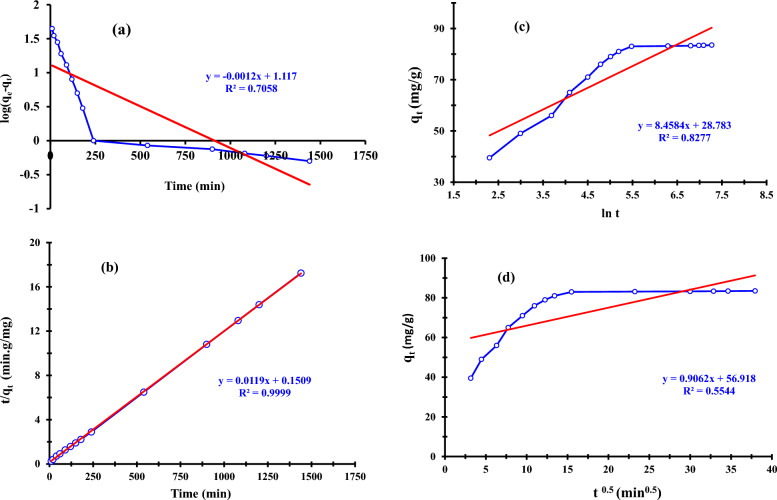
Kinetic models: **a** Pseudo-first-order, **b** Pseudo-second-order, **c** Elovich, and **d** Intraparticle diffusion plots of phosphorous removal by ZnO NPs

Kinetics informs us of the adsorption process' regulating processes and aids in creating useful mathematical models that capture the interactions [23]. The rate-limiting phase of phosphorous adsorption by ZnO NPs was examined by fitting the experimental data to four kinetic models, including pseudo-first and second-order, Elovich, and intraparticle diffusion kinetic models. The slope of a graph of log (q_e_ − q_t_) *vs* time t is used to calculate the value of K_1_, as shown in Fig. 10a. According to Table 3, the correlation coefficient value for the three ZnO NPs was 0.7058, indicating that this model is unable to explain the experimental data. ZnO NPs experimentally determined adsorption capacity of 84 mg/g, derived from Fig. 10b, which was comparable to the calculated value of 84.3 mg/g obtained using the pseudo-second-order method. The results shown in Table 3 demonstrate that the pseudo-second-order kinetic model adequately described the experimental data, as indicated by the correlation coefficient (R^2^ = 0.9999). The good fit of P sorption kinetics to the pseudo-second-order model suggests that the rate-limiting phase in the sorption of P ions onto ZnO NPs is likely governed by chemisorption involving valence forces through the sharing or exchange of electrons between the NPs surface and P ions [80]. Less linearity is evident in the regression coefficient R^2^ for the Elovich and intraparticle diffusion models, which were determined to be 0.8277 and 0.5544, respectively, in Fig. 10c and d. It can be concluded that the models proposed by Elovich and intraparticle diffusion are not applicable for P ions removal by ZnO NPs based on the **R**^**2**^ value obtained by the second-order kinetic model.

**Table 3:** Kinetic parameters for P ions onto ZnO NPs adsorbent

Kinetics models			
Pseudo-first-order	Pseudo-second-order	Elovich	Intraparticle diffusion
q_e_ = 13	q_e_ = 84.3	α = 0.25 × 10^3^	K_int_ = 0.9
K_1_ = 0.0028	K_2_ = 0.000931	β = 0.11	C = 56.9
R^2^ = 0.7058	R^2^ = 0.9999	R^2^ = 0.8277	R^2^ = 0.5544

#### Adsorption isotherms

**Fig. 11:**
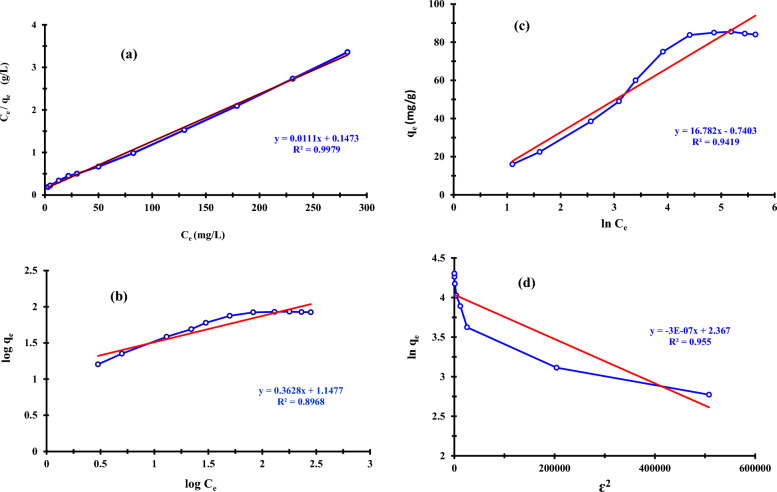
Adsorption isotherms: **a** Langmuir, **b** Freundlich, **c** Temkin and **d** Dubinin-Radushkevich isotherms plots of phosphorous removal by ZnO NPs

At 27 °C and pH 3.0, an equilibrium analysis was conducted to assess the experimental data to calculate the adsorption capacity of the adsorbents. Table 4 provides a list of the calculated and experimental results. A straight line is produced by plotting the value of C_e_/q_e_ vs C_e_ along with the related Langmuir constants for removing P ions by ZnO NPs, as illustrated in Fig. 11a. The outcomes showed that the adsorption process fit the Langmuir model well, with R^2^ values of 0.9979 suggesting that phosphorous ions would be adsorbed homogeneously by all adsorption active sites with identical affinity. The elimination of phosphorus was determined to have a favorable separation factor, or R_L_ value, of 0.075 using the Langmuir model. Figure 11b depicts the Freundlich adsorption isotherm model's log q_e_ vs. log C_e_ plot. The 1/n values in this study are between 0 and 1, indicating a typical process of phosphorus adsorption onto ZnO NPs adsorbent. Moreover, as shown in Table 4, the n value, which ranges between 1 and 10, implies a good adsorption process. The Freundlich isotherm was not the best fit for the sorption process of phosphorus onto the surface of the ZnO NPs, as evidenced by the R^2^ values of 0.8968. Phosphorous ions are thus removed from the surface of ZnO NPs with certain heterogeneity inactive spots. The heat of sorption corresponds to a physical process because the predicted Temkin parameters A and B values from Fig. 11c and stated in Table 4 were determined to be 0.96 L/g and 16.78 J/mol, respectively.

**Table 4:** Isotherm parameters for P ions removal onto ZnO NPs adsorbent

Isotherm models			
Langmuir	Freundlich	Temkin	Dubinin-Radushkevich
q_m_ = 89.8	K_F_ = 14	A = 0.96	q_m_ = 68.7
K_L_ = 0.076	n = 2.8	B = 16.8	E = 0.564
R_L_ = 0.075		b_T_ = 0.148	
R^2^ = 0.9979	R^2^ = 0.8968	R^2^ = 0.9419	R^2^ = 0.7848

Additionally, the b_T_ values of 0.148 kJ/mol demonstrated that the removal was accomplished through a physisorption mechanism [84]. The plot of ln q_e_ versus ε^2^ in Fig. 11d displays the D-R parameters; the slope of the plot displays β (mol^2^/J^2^), and the intercept displays q_m_ (mg/g). Each sorbate molecule's free energy (E) of sorption when moving from infinity in the solution to the surface of the solid was found to be 0.56 kJ/mol. This finding confirms that the physical sorption process of P onto ZnO NPs adsorbents, as indicated in Table 4, occurs.

The Langmuir isotherm model has the highest R^2^ values, according to a comparison of the values in Table 4. The Langmuir isotherm was therefore the one that fit sorption the best. The Temkin heat of adsorption constant (B) values and b_T_ for nano-adsorbents, along with the free energy (E) of sorption per molecule of the sorbate in the Dubinin-Radushkevich model, supported the conclusion that the removal happened via physisorption process.

#### Thermodynamics study

**Fig. 12:**
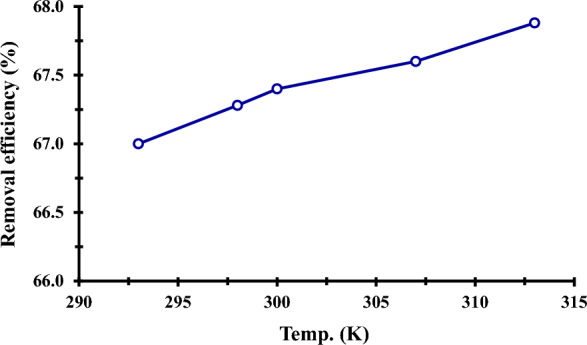
Effect of temperature (K) in adsorption of phosphorous by ZnO NPs. (250 mg/L initial P ions concentration, 0.040 g adsorbent dose, temperature 27 °C, 24 h)

The effects of temperature on phosphate adsorption onto ZnO NPs are depicted in Fig. 12 with temperatures ranging from 296 to 313 K, an initial phosphorus concentration of 250 mg/L, pH 3, and a fixed adsorbent dosage of 40 mg/L. The gradual rise in phosphorus adsorption capacity with temperature was detected, indicating that phosphorus adsorption onto ZnO NPs was an endothermic reaction, as was previously seen with Zhen Luo [14]. The possibility of the adsorption process and the spontaneous character of the adsorption are both confirmed by the negative values of ΔG (ΔG < 0) in Table 5 [85]. Additionally, the adsorption process appears more advantageous at higher temperatures, as seen by the decrease in the negative value of ΔG with an increase in temperature [16, 86, 87]. When ΔH has a positive value, the adsorption process is endothermic. Finally, the positive values of ΔS show unmistakably that during the phosphorus adsorption onto the ZnO NPs, the randomness at the solid-solution interface increased [88, 89]. This may be caused by the phosphorus's increased mobility [90].

**Table 5:** Thermodynamic parameters for P ions adsorption by ZnO NPs adsorbent

Temp. (K)	K_d_ (L/mg)	ΔG (kJ/mol)	ΔH (kJ/mol)	ΔS (KJ/mol)
296	0.015	−0.037	+ 1.46	+ 4.7
298	0.078	−0.069		
300	0.033	−0.083		
307	0.042	−0.110		
313	0.055	−0.143		

### A comparative study with other adsorbents

This work focused on the high efficiency of ZnO NPs, which surpassed many earlier studies employing numerous nano adsorbents for removing phosphorous from aqueous solutions. ZnO NPs have not been widely employed as an effective adsorbent in removing phosphorous from aqueous solutions (Table 6). Compared to other nano-adsorbents, the produced ZnO NPs are thought to have an exceptional capacity to adsorb P ions and remove them from an aqueous solution. Therefore, it may be concluded that a larger percentage of ZnO NP adsorbents are sorbent at high P concentrations. As is evident, ZnO NPs used in this study outperform many other adsorbents mentioned in the literature. High specific surface area ZnO NPs were credited with the produced nanomaterials' superior adsorption capacity. The outcome showed that the sorption process required a considerably lower dosage of ZnO NPs.

**Table 6:** Maximum adsorption capacities of phosphorus onto various adsorbents

NO	Adsorbents	Capacity of P (mg/g)	References
1	TiO_2_ nanoparticles AL_2_O_3_ nanoparticles Fe_3_O_4_ nanoparticles	28.3 24.4 21.5	[91]
2	Hydroxy-aluminum pillared bentonite	12.9	[88]
3	Iron oxide-coated Fly Ash	8.95	[92]
4	SnO_2_ nanoparticles WO_3_ nanoparticles	21.5 19.0	[16]
5	Ag nanoparticles-loaded activated carbon	4.5	[93]
6	Bayoxide	9.1	[94]
7	Hydrated ferric oxide nanoparticles	12.86	[7]
8	Magnetite-enriched particles	6.4	[95]
9	Magnetite-based nanoparticles	5.2	[96]
10	Biochar derived from Sewage Sludge	4.8	[97]
11	ZSFB-composite fiber	4.18	[98]
12	Hollow-magnetic -Fe_3_O_4_	11.95	[99]
13	NaCl modified zeolite	6.67	[100]
14	ZnO particles	53.4	[14]
15	ZnO nanoparticle	89.8	This Study

### Recovery of phosphorus and reusability of ZnO NPs

**Fig. 13:**
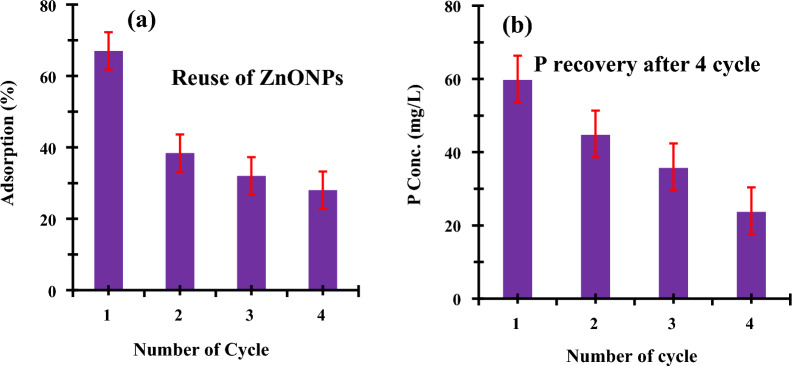
Phosphorous removal from water: Recovery and reuse of ZnO NPs. The same nanoparticles were washed and reused for 4 times. (a) Reuse of ZnO NPs for 4 times. For each experiment fresh P-solution was used. (b) Recovery of phosphorous after washing with alkaline treatment

After regeneration, the effectiveness of using ZnO NPs repeatedly for P removal was looked at and displayed in Fig. 13. According to the findings (Fig. 13a), cycles 1–4 had average removal efficiencies of 38, 32, and 28%, respectively. The loss of functional groups, insufficient desorption, washing under alkaline conditions, or complex compounds that may have occluded the interaction sites in ZnO NPs could all contribute to the decline in adsorption effectiveness. This finding suggests that ZnO NPs can be used again for phosphorus removal from water for a finite period. ZnO nanoparticles are subjected to alkaline conditions in cycles, and tend to release the adsorbed phosphorus from the surface into the liquid phase. Figure 13b shows the emission of phosphorus in proportions.

Measurements of the experimental test procedure were carried out in triplicates to confirm the accuracy and consistency of the generated data according to the criteria of repeatability and validation [82, 101–104, 107, 109]. In four cycles, the same nanoparticles underwent cleaning and reuse. (a) Reusing ZnO nanoparticles four times. Each experiment used a brand-new P-solution. (b) Phosphorus recovery with alkaline treatment and washing.

### Antimicrobial study

**Fig. 14:**
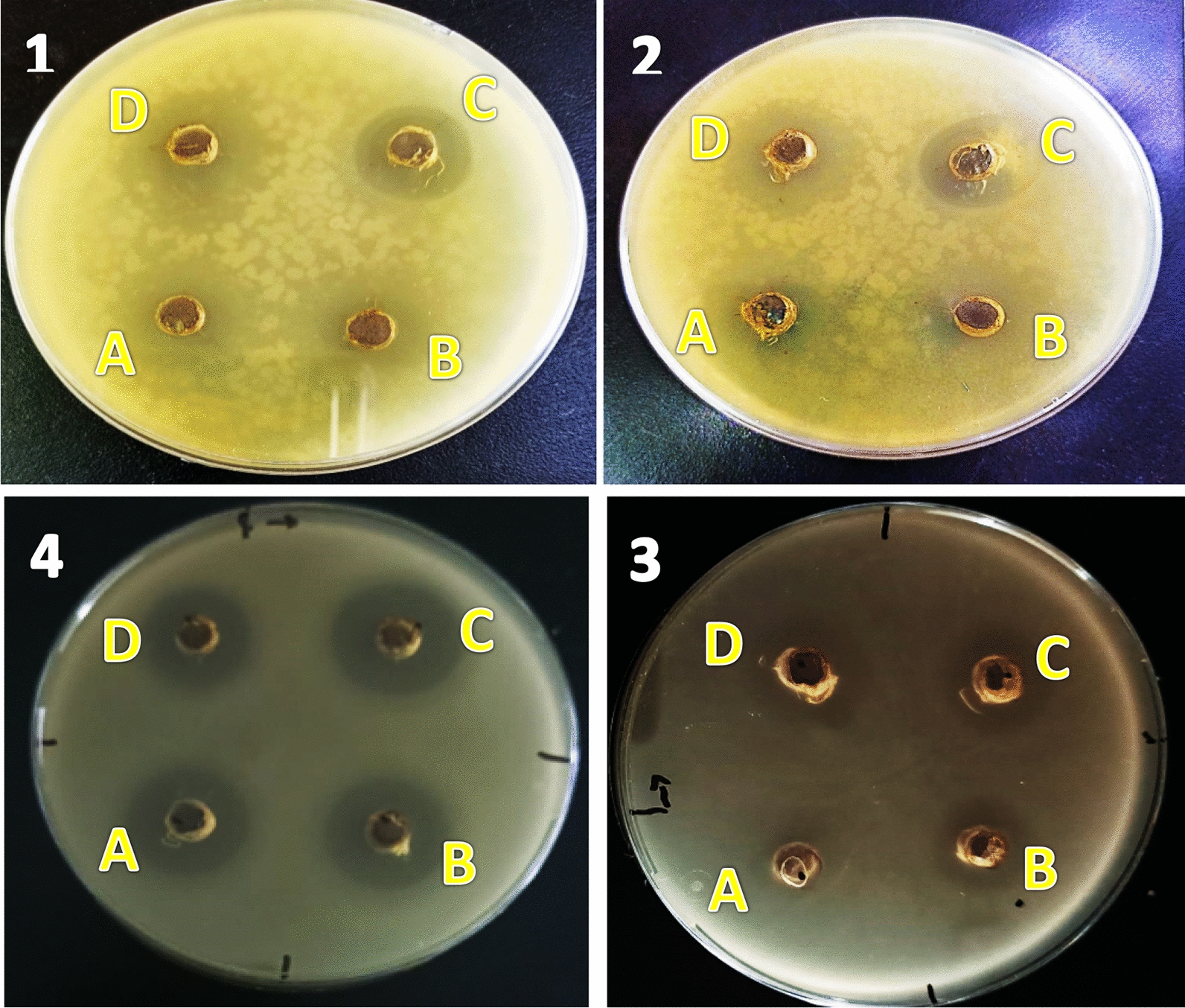
Antimicrobial activity of ZnO NPs solutions against; **1** *E. coli* **2** *S. aureus* **3** *E. faecalis* **4** *S. typhimurium* for representing bacteria. The letters A, B, C, and D refer to the concentration of solutions (A = 50, B = 100, C = 150, and D = 200 μg/mL)

**Fig. 15:**
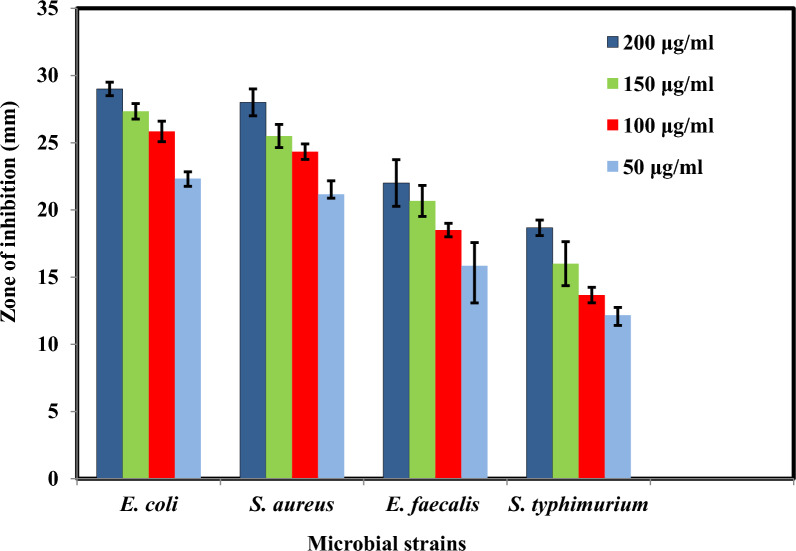
Antibacterial activities of the biosynthesis ZnO NPs *E.coli*, *S. aureus*, *E. faecalis*, and *S. typhimurium*. ZnO NPs showed more inhibitory activity against bacteria Gram-negative. *E. coli* exhibited the highest inhibition zone

The minimum inhibitory concentration (MIC) values (mean ± standard deviation; n = 3) for the biosynthesized ZnO NPs were used to assess their antibacterial activity [108]. The results are shown in Table 7. The results demonstrated inhibitory zones against Gram-negative bacteria *S. typhimurium* and *E. coli*, *E. faecalis*, and *S. aureus* after treatment with ZnO NPs at various doses of 50, 100, 150, and 200 μg/mL. (Gram-positive bacteria). The ZnO NPs in Fig. 14 showed good potentiality against all tested microorganisms. The values of the inhibition zone are shown in Fig. 15, and the variation in the zone depends on the concentration of ZnO NPs used in the antibacterial activity. The correct diffusion of nanoparticles in the agar medium has also consistently boosted growth inhibition. *E. coli* (MIC MIC 29 ± 0.5 mm), *S. aureus* (MIC 26.8 ± 0.29 mm), *E. faecalis* (MIC 22 ± 1.7 mm), and *S. typhimurium* (MIC 18.7 ± 0.58 mm) were shown to be the species most sensitive to the nanoparticles. According to Shaban et al. [105], Gram-positive bacteria have a thick peptidoglycan cell wall, while Gram-negative bacteria have cell walls primarily composed of lipopolysaccharides, which provide a less effective defense against the passage of hazardous species into the cytoplasm. Therefore, the presence of an inhibitory zone demonstrates that the mechanism of ZnO NPs activities, which include membrane rupture with a high rate of surface oxygen species multiplication and ultimately result in pathogen mortality, is involved [65].

**Table 7:** Antibacterial activity of ZnO NPs, as determined by minimum inhibitory concentration (MIC) value (mean ± standard deviation; n = 3)

Microorganisms	Gram reactive	Inhibition zone (mm)	
		ZnO NPs 50 μL from 100 μg/L	Cefotaxime 50 μL from 150 μg/L
*E.coli*	−ve	29 ± 0.5	27 ± 2.08
*S.aureus*	+ ve	26.8 ± 0.29	27 ± 0.99
*E. faecalis*	+ ve	22 ± 1.7	20 ± 1.73
*S. typhimurium*	−ve	18.7 ± 0.58	21 ± 2.51

**Fig. 16:**
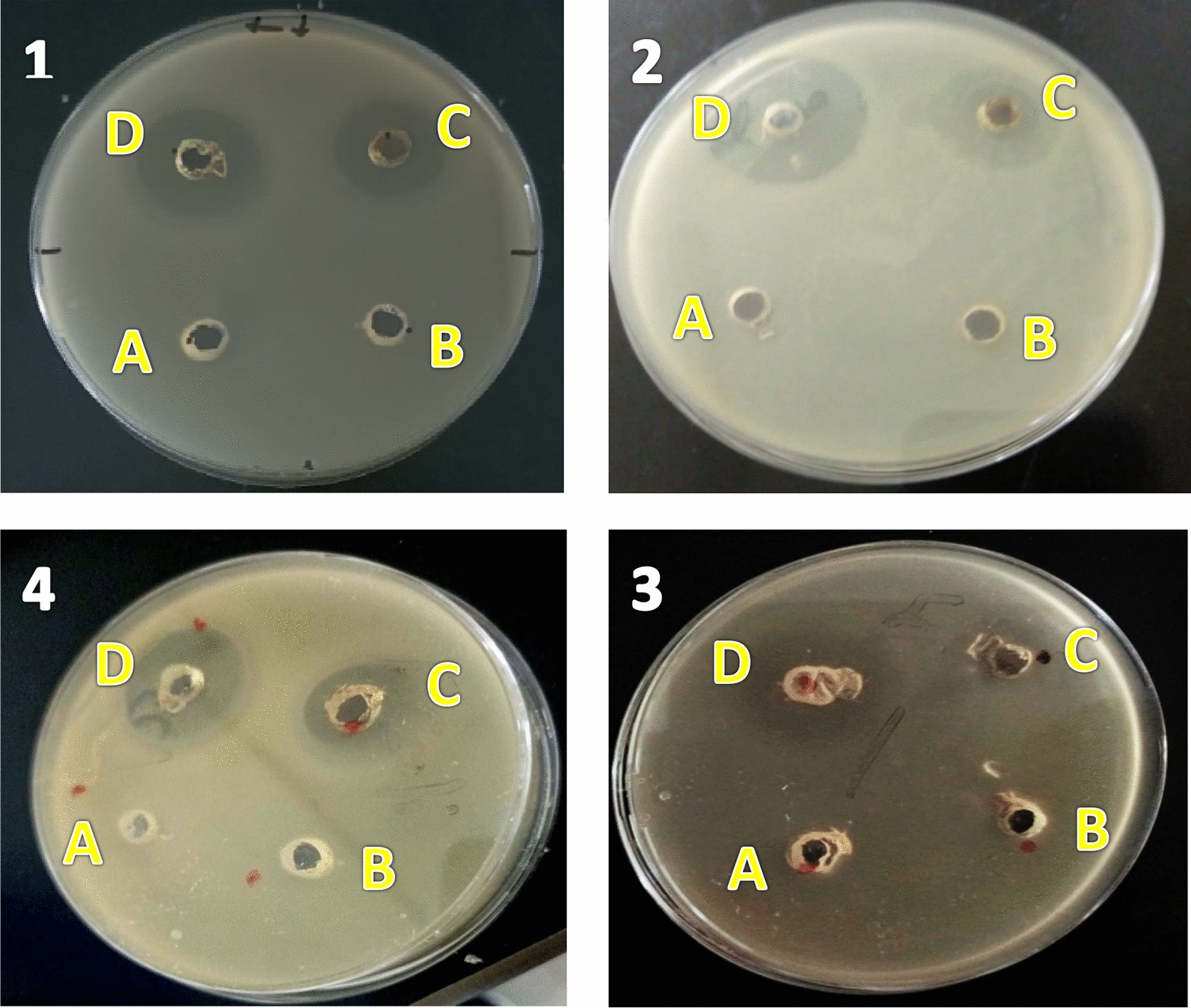
Antimicrobial activity of (100 μg/mL) ZnO NPs against **1** *E.coli* **2** *S. aureus* **3** *E. faecalis and S. typhimurium* (**4**), the letters A, B, C, and D refer to DMSO and *onion* extract, 100 μg/L ZnO NPs, 150 μg/L of Cefotaxime, respectively

Comparing 50 μL of (100 μL/mL) ZnO NPs with 50 μL of (150 μg/L) Cefotaxime, DMSO, and *Onion* extract is shown in Fig. 16. The results indicated that the as-synthesized ZnO NPs have greater antibacterial action than standard Cefotaxime, which was utilized as a positive control. In contrast, the controlled wells supplemented with onion extract and DMSO failed to show any inhibitory zone. Gram-negative bacteria were shown to be more vulnerable to prepared nanoparticles than Gram-positive bacteria, as evidenced by the highest inhibition zone against *E. coli* and *S. aureus* and the order of *E. faecalis*, *S. typhimurium*, and *E. Faecalis*. However, *S. typhimurium* had a smaller inhibitory zone, showing that the structural variations in the cell membrane of bacteria account for the changes in their antibacterial activity [106]. In conclusion, the antimicrobial activity showed that ZnO NPs were more hazardous to bacterial cells and may be more effective than standard antibiotics at inactivating some human diseases.

## Conclusion

The first demonstration of using onion extract as a powerful oxidizing/reducing chemical agent to produce ZnO NPs used a green, unique, and ecologically acceptable method for producing extremely crystalline ZnO NPs. ZnO NPs were characterized using XRD, UV–Vis spectroscopy, FTIR, Raman, SEM, EDX, and TEM. ZnO NPs were used in this work to successfully remove a considerable amount of phosphorus from water. When phosphorus concentration was initially concentrated to 250 mg/L, the maximum adsorption capacity was 89.8 mg/g. The results indicated that a chemisorption process controlled P sorption, and a better match might be obtained by using the kinetics model of pseudo-second-order equations. Additionally, the sorption equilibrium results followed the Langmuir models in the concentration ranges examined. The spontaneous, endothermic, temperature-dependent sorption of phosphorus ions on ZnO NPs. The results showed that the average removal efficiencies of the phosphorus recovery and reusability of ZnO NPs for cycles 1–4 were 38, 32, and 28%, respectively. This finding shows that ZnO NPs can be utilized to temporarily remove phosphorus from water once more. The biosynthesized ZnO NPs were also considered for antibacterial uses. ZnO NPs exhibit superior antibacterial effects against Gram-negative and Gram-positive bacteria when compared to the common antibiotic Cefotaxime. Following *E. faecalis* and *S. typhimurium*, *E. coli,* and *S. aureus* displayed the highest degrees of inhibition, illustrating the ZnO selective influence on biological systems.

## Data Availability

All data generated or analyzed during this study are included in this article and the raw data is available from the corresponding author if requested.
